# Factors Explaining the Coincidence of Smoldering Multiple Myeloma and Primary Biliary Cholangitis: A Case Report

**DOI:** 10.7759/cureus.26830

**Published:** 2022-07-13

**Authors:** Mirna El Dirani, Julius M Nagaratnam, Samer Kholoki

**Affiliations:** 1 Internal Medicine, Saint James School of Medicine, Chicago, USA; 2 Internal Medicine, Avalon University School of Medicine, Chicago, USA; 3 Internal Medicine, La Grange Memorial Hospital, Chicago, USA

**Keywords:** autoimmune cirrhosis, autoimmune disease and cancer, multiple myeloma prognosis, smoldering multiple myeloma, primary biliary cirrhosis (pbc)

## Abstract

To date, there have been nine reported instances of coinciding smoldering multiple myeloma (SMM) and primary biliary cholangitis (PBC). The term SMM was coined in 1980 to describe low-severity multiple myeloma cases, a hematologic neoplasia that involves the malignant proliferation of plasma cells. PBC is an autoimmune disorder targeting the intrahepatic bile ducts and is characterized by elevated anti-mitochondrial antibodies and often resulting in autoimmune liver cirrhosis. Currently, there is no plausible rationale for the coincidence of SMM and PBC in patients. This report investigates the relationship between SMM and PBC in a Hispanic 49-year-old female residing in the United States and attempts to determine the possible genetic and biochemical causes of this coincidence.

## Introduction

Multiple myeloma (MM) is the second most prevalent hematologic neoplasia [1], with a worldwide incidence of 160,000 cases and mortality of 106,000 patients in 2018 [2]. In 1980, the term smoldering multiple myeloma (SMM) was used to describe MM without significant symptoms, noted in 8% to 20% of MM cases [3]. A case was classified as SMM if there was no evidence of end-organ damage, no disease progression for five years, an M-protein level exceeding 30 g/L, and bone marrow plasmacytosis exceeding 10% [3].

Primary biliary cholangitis (PBC), however, is a rare autoimmune disorder with an estimated 12-year prevalence in the United States of 29.3 per 100,000 individuals [4]. A patient is diagnosed with PBC based on the clinical presentation, detectable anti-mitochondrial antibody (AMA) levels, abnormal liver function test (LFT) parameters for six months [5,6], and histological findings showing chronic nonsuppurative inflammation of interlobular and septal bile ducts [5].

Cases of coinciding SMM and PBC are rare, and only nine cases have been documented in the literature [7], with the last case reported in 2008 [7]. Of the nine cases, only one case demonstrated immunoglobulin (Ig)A predominance in 1993 [7,8], while the remaining cases demonstrated IgG predominance and Bence-Jones proteinuria [7].

## Case presentation

In 2011, a 49-year-old Hispanic female presented to her primary care physician’s clinic complaining of generalized fatigue and pain in the extremities. Following a comprehensive blood count and a bone marrow biopsy (BMB) which demonstrated 10% plasmacytosis, she was diagnosed with SMM on September 8, 2011. Since this visit, she did not have the presenting findings that met the CRAB and SLiM criteria [9], nor did she become more symptomatic.

In 2017, the patient presented to the emergency department complaining of right upper quadrant pain, nausea without vomiting, and generalized fatigue. A comprehensive metabolic panel demonstrated elevated liver enzymes (aspartate transaminase (AST), alanine transaminase (ALT), and alkaline phosphatase), which was followed by an abdominal computed tomography (CT) which showed ascites, splenomegaly, and gastric varices. A liver biopsy performed on March 3, 2017, demonstrated findings suggestive of PBC and stage III fibrosis, suggesting autoimmune hepatitis (AIH). Since her diagnosis, the patient’s transaminases continued to be elevated (AST ranging between 73 U/L and 144 U/L, ALT ranging between 42 U/L and 89 U/L). The patient was placed on an evolving medication regimen including prednisone 15 mg once daily, myfortic, azathioprine 50 mg twice daily, and cyclosporine 50 mg twice daily.

After being diagnosed with PBC and SMM simultaneously, the patient had a history of frequent hospitalizations associated with complications linked to having both coinciding conditions. On June 1, 2017, she underwent a laparoscopic cholecystectomy after being diagnosed with gallbladder cholesterolosis without cholelithiasis, after which she was placed on long-term ursodiol 1,200 mg once daily.

She also had numerous hospitalizations for recurrent transient small bowel obstruction (SBO), with her first episode occurring in 2017, diagnosed on an abdominal CT which showed distal SBO secondary to postoperative adhesions. The patient was again hospitalized for symptoms suggestive of SBO on March 23, 2019, with an abdominal CT on admission demonstrating SBO due to adhesions with dilation of several loops of the distal ileum. Her condition was managed with the placement of a nasogastric (NG) tube and supportive management, and she was discharged a day later. The patient was hospitalized for numerous other episodes of SBO-related symptoms on June 26, 2021, August 21, 2021, October 19, 2021, and November 17, 2021, all of which were resolved with the placement of an NG tube.

**Figure 1:**
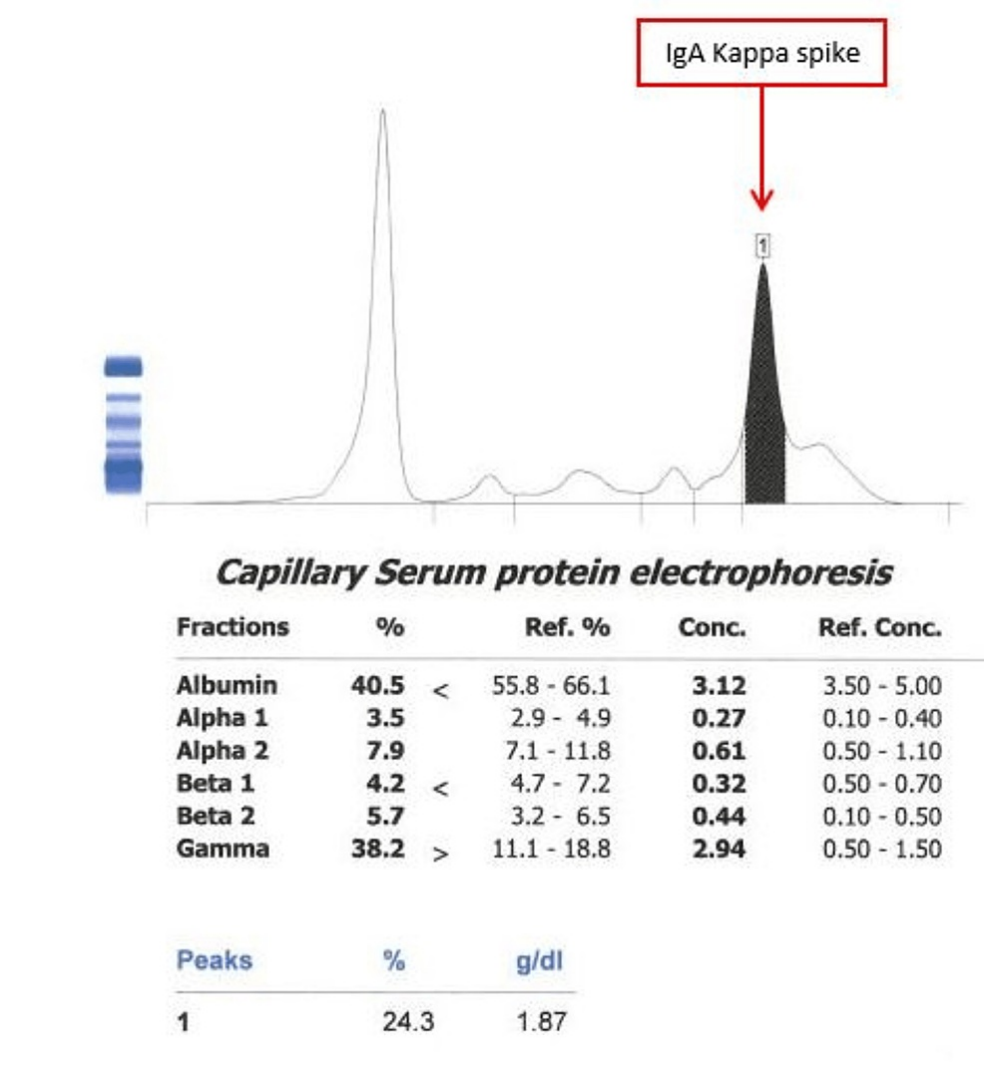
Serum protein electrophoresis performed in 2020 demonstrating immunoglobulin A predominance.

A fluorescence in situ hybridization (FISH) analysis done on July 8, 2020, revealed a chromosome 13 monosomy, and a serum protein electrophoresis (SPEP) demonstrated a monoclonal IgA kappa level of 1.9 g/dL, light chains kappa level of 10.5 mg/dL, and lambda level of 2.51mg/dL, ruling the patient’s SMM as IgA predominant (Figure 1). A repeat BMB done on September 3, 2020, found plasmacytosis had increased to 15%, as well as a cytogenetic analysis revealed a t(4;14) translocation and 1q21 microdeletion which were deemed high-risk cytogenetic findings.

The patient was finally admitted to the hospital on February 7, 2022, for elevated bilirubin (29.6 mg/dL) and ammonia (110 µg/dL), as well as increasing lower extremity edema and abdominal girth due to progressing ascites. Magnetic resonance cholangiopancreatography demonstrated a large amount of ascites and cirrhotic liver with evidence of portal hypertension. Dual diuretic therapy did not relieve the patient’s ascites. Consequently, an ultrasound-guided paracentesis of the right lower quadrant was performed, obtaining 4,600 mL of clear yellow fluid. During this visit, discussions regarding end-of-life care were initiated because the patient’s condition declined dramatically.

## Discussion

There are several similarities between this case and previously documented cases of coinciding PBC and SMM. Similar to previous cases, this patient demonstrated hyperimmunoglobulinemia, secondary to extensive production from proliferating plasma cells. In addition, the patient’s signs and symptoms were relatively similar to previously documented cases. Generalized fatigue and extremity pain align with the traditional symptoms suggestive of MM [10], and the presence of right upper quadrant pain, nausea, ascites, and hepatosplenomegaly are traditional signs and symptoms of PBC [11].

However, the uniqueness of this case stems from the fact that this patient is one of two reported cases of SMM with IgA kappa predominance. After IgG predominance, IgA predominance is the second most common form of MM, with 21% of patients being affected according to a study performed by Mayo Clinic in 2003 [12].

Moreover, unlike previously published cases, this patient’s liver biopsy at the time of diagnosis was far more advanced, demonstrating more extensive fibrosis at a younger age. Previous studies have shown that patients with PBC and overlapping AIH are likely to have more rapidly progressing liver necrosis and fibrosis [13-15]. The prevalence of PBC-AIH overlap syndrome varies between 2% and 20% [16] of patients with a single diagnosis of PBC or AIH. None of the previously published case studies with coinciding PBC and MM demonstrate the additional phenomenon of PBC-AIH overlap syndrome.

A plausible explanation for the simultaneous onset of SMM and PBC may be explained by increased plasma cells hypersecreting pathogenic antibodies such as AMA and anti-PDC-E2 [7]. The severity of this patient’s liver cirrhosis may be explained by IgA dimerizing with anti-PDC-E2 to form IgA-anti-PDC-E2, which is significantly associated with PBC progression [17].

Other genetic and biochemical factors that may contribute to the simultaneous onset of SMM and PBC include the presence of a t(4;14) translocation, found in approximately 15% of MM cases, which results in FGFR3 overexpression. The occurrence of a chromosome 1q21 microdeletion is found in 40% of MM patients, which results in overexpression of oncogenes CKS1B and SKP2 [18]. Previous studies have also suggested that PBC and MM have an overlapping single-nucleotide polymorphism affecting the human leukocyte antigen-DQB1 [19,20]. A quantitative analysis of these genetic and biochemical findings may help determine their significance in the association between PBC and MM.

## Conclusions

The simultaneous occurrence of PBC and SMM is an exceptional example of an association between an autoimmune disorder and neoplasia. Future quantitative analysis would be useful in determining the significance of the factors involved in the association between SMM and PBC. This is imperative in further understanding the dynamic interplay of both conditions and in improving the education and management of patients.
